# Supplementary material for: Identification of photoperiod-induced specific miRNAs in the adrenal glands of Sunite sheep (Ovis aries)
Source: Front Vet Sci. 2022 Jul 22;9:888207. doi: 10.3389/fvets.2022.888207 (PMC9354845; doi:10.3389/fvets.2022.888207)
Supplement: Supplementary file 1 [file Data_Sheet_1.ZIP › Supplementary file/miRDeep2 file.zip!/miRDeep2 file/Novel_Structure/SPLP42A3/Novel_5.pdf]

Provisional ID	:	NC_019476.2_30671
Score total	:	0.1
Score for star read(s)	:	-1.3
Score for read counts	:	0
Score for mfe	:	-0.1
Score for randfold	:	1.6
Score for cons. seed	:	
Total read count	:	105
Mature read count	:	105
Loop read count	:	0
Star read count	:	0

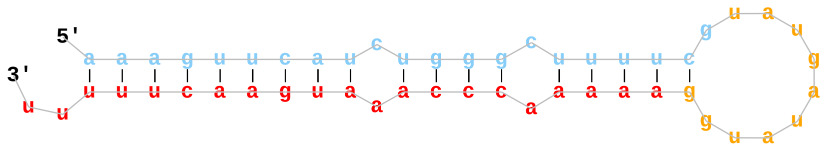

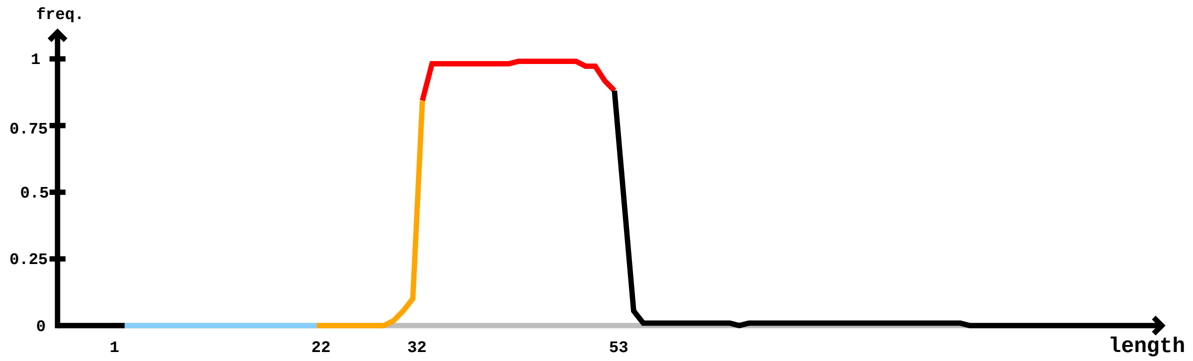


```
          Star                         Mature
5'- accaaaaaguucaucugggcuuuucguaugauauggaaaaacccaaaugaacuuuuugaccaacccaauaccuauuuguaugcuucuccugcucaguugcucagugaugucug   -3'   exp
    ..((((((((((((.((((.(((((..........))))).)))).)))))))))))).........((((......))))((.((....((......)).....)).))...    reads      mm      sample
    .................................uggaaaaacccGaaugaacu............................................................    2          1       seq
    ..................................ggaaaaacccaaaugaacuuu..........................................................    1          0       seq
    ..................................Ugaaaaacccaaaugaacuuu..........................................................    1          1       seq
    ..................................ggaaaaacccaaaugaacuuuu.........................................................    2          0       seq
    ...................................gaaaaacccGaaugaacuuu..........................................................    1          1       seq
    ...................................gaaaaacccaGaugaacuuu..........................................................    2          1       seq
    ...................................gaaaaacccGaaugaacuuuu.........................................................    1          1       seq
    ...................................gaaaaacccaGaugaacuuuuu........................................................    1          1       seq
    ....................................aaaaacccGaaugaacuuu..........................................................    1          1       seq
    ....................................aaaaacccaGaugaacuuuu.........................................................    1          1       seq
    ....................................Gaaaacccaaaugaacuuuuu........................................................    3          1       seq
    ....................................aaaaacccaGaugaacuuuuu........................................................    27         1       seq
    ....................................aaaaacccaaaugaacuuuuuA.......................................................    2          1       seq
    ....................................Caaaacccaaaugaacuuuuug.......................................................    3          1       seq
    ....................................aaaaacccGaaugaacuuuuug.......................................................    2          1       seq
    ....................................Gaaaacccaaaugaacuuuuug.......................................................    6          1       seq
    ....................................aaaaCcccaaaugaacuuuuug.......................................................    1          1       seq
    ....................................aaaaacUcaaaugaacuuuuug.......................................................    24         1       seq
    ....................................aaaaGcccaaaugaacuuuuug.......................................................    2          1       seq
    ....................................aaaaacccaaaugaacuuuuug.......................................................    8          0       seq
    ....................................aaaaacccaaaugaacuuuuuga......................................................    1          0       seq
    .....................................aaGacccaaaugaacuuuuu........................................................    1          1       seq
    .....................................aaaacUcaaaugaacuuuuug.......................................................    10         1       seq
    .....................................aaaacccaaaugaacuuuuuga......................................................    4          0       seq
    ..............................................augaacuuuuugaccaacccaaA............................................    1          1       seq
    ......................................................................ccuauuuguaugcuucuccugcu....................    1          0       seq
```
